# Dataset on the essential role of Anks1a in Xenopus laevis morphogenesis: evidence from morpholino knockdown and mRNA rescue experiments

**DOI:** 10.1016/j.dib.2026.113283

**Published:** 2026-09-23

**Authors:** Vijay Kumar, Shiv Kumar, Unjoo Lee, Jaebong Kim

**Affiliations:** a Department of Biochemistry, Institute of Cell Differentiation and Aging, College of Medicine, Hallym University, Chuncheon, Gangwon-Do, 24252, Republic of Korea; b Department of Electrical Engineering, Hallym University, Chuncheon, Gangwon-Do, 24252, Republic of Korea; c Department of Biochemistry, College of Medicine, Konyang University, 158, Gwanjeodong-ro, Seo-gu, Daejeon 35365, Republic of Korea; d Scintillon Institute, 6888 Nancy Ridge Dr. San Diego, CA-92121, USA

**Keywords:** Anks1a, ODIN, Xenopus laevis, Morpholino knockdown, mRNA rescue, Embryonic morphogenesis, Developmental defects

## Abstract

Ankyrin Repeat and Sterile Alpha Motif Domain Containing 1A (ANKS1A), also known as ODIN, is a multifunctional adaptor/scaffolding protein that regulates receptor tyrosine kinase signaling and intracellular protein trafficking. While morpholino (MO)-mediated knockdown of *Anks1a* in Xenopus has been shown to impair ciliogenesis and motile cilia function, its role in early embryonic morphogenesis remains largely unknown. Here we report morphological phenotype images and quantification from morpholino-mediated *Anks1a* knockdown and mRNA rescue experiments in *Xenopus laevis* tadpoles. The data document dose-dependent defects in head size, melanocyte distribution, reduced or malformed cardiac region, and gut coiling, together with partial phenotypic rescue upon co-injection of mouse *ANKS1A-VN* mRNA. After *Xenopus anks1a* morpholino (*xanks1a-*MO), 77.6% of embryos were Severe (*n* = 85); co-injection with mouse *ANKS1A-VN* mRNA gave 16% Normal, 66% Mild and 18% Severe (*n* = 100). Raw image files and scoring data are available in the Zenodo repository (DOI: 10.5281/zenodo.21912238).

Specifications Table

**Table:** 

Subject	Biology
Specific subject area	Developmental Biology, Molecular Biology, Biochemistry
Type of data	Images (morphological phenotypes), Table, Figures
Data collection	One-cell-stage Xenopus laevis embryos were microinjected with *xanks1a*-MO (75, 100 or 150 ng in 2 nl) alone or co-injected with 500 pg *ANKS1A-VN* mRNA (1 nl; total 3 nl). Non-injected and 10% DMSO vehicle-injected embryos were included as controls. Embryos were cultured to stages 35–36 and 42–43 and scored as Normal, Mild or Severe.
Data source location	Institution: Hallym University, Chuncheon, Gangwon-Do, Republic of Korea
Data accessibility	Repository name: Zenodo Data identification number: 10.5281/zenodo.21912238 Direct URL to data: https://doi.org/10.5281/zenodo.21912238
Related research article	Ryu, H., Lee, H., Lee, J., Noh, H., Shin, M., Kumar, V., Hong, S., Kim, J., & Park, S. (2021). The molecular dynamics of subdistal appendages in multi-ciliated cells. *Nature Communications*, 12, 612.

## 1 Value of the Data

• The dataset contains stage-wise RT-PCR expression profiles of *anks1a* (stages 1–40), dose-response morphological images of *Xenopus laevis* embryos injected with *xanks1a-*MO (75 ng, 100 ng, or 150 ng), and corresponding images from co-injection rescue experiments with *ANKS1A-VN* mRNA, together with quantified phenotypic classification (Normal, Mild, and Severe) at stages 35–36 and 42–43.
• Live-embryo image series document specific morphological features under the tested conditions, including eye size, cranial melanocyte distribution, cement-gland/oral region morphology, reduced or malformed cardiac region and branchial regions, segmented myotomes, gut coiling, and tail/caudal-fin structure, presented in lateral and ventral/anterior views.
• The data complement the previously published study on Anks1a function in multi-ciliated cells [1] by supplying whole-embryo morphogenetic readouts obtained with the same MO reagent and rescue approach.
• Researchers working on vertebrate embryonic development, neural-crest derivatives, cardiac morphology, gut morphogenesis [2], or MO-based loss-of-function studies in Xenopus can reuse the image sets, scoring criteria, and experimental parameters for direct comparison or methodological reference.

## 2 Background

This dataset was compiled in the context of ongoing investigations into the multifunctional adaptor protein Anks1a (also known as ODIN) and its roles in receptor tyrosine kinase signaling, intracellular trafficking, and ciliogenesis [3]. Prior work had established that MO-mediated knockdown of *anks1a* in *Xenopus* impairs ciliogenesis and motile cilia function in multi-ciliated cells [1]. The present experiments applied the same translation-blocking *xanks1a*-MO oligonucleotide, alone or co-injected with 500 pg mouse *ANKS1A-VN* mRNA, to one-cell-stage *Xenopus laevis* embryos. Embryos were grown to tadpole Nieuwkoop and Faber (NF) stages (35–36 and 42–43) and examined for whole-embryo morphological phenotypes across a dose range (75–150 ng MO) [4,5]. The data article supplies the accompanying stage-wise RT-PCR expression profiles, dose-response image series, and quantified phenotypic classes (Normal/Mild/Severe) that were generated during these morpholino and rescue trials.

## 3 Data Description

The dataset comprises primer and MO sequences (Table 1), stage-wise RT-PCR profiles, live-embryo image series from *xanks1a* MO knockdown and mRNA rescue, and the phenotypic scoring used for Fig. 3. Unprocessed images and the numerical scoring table are deposited at Zenodo (https://doi.org/10.5281/zenodo.21912238) [6].

**Table 1:** Sequences of primers used for RT-PCR analysis of *Odc1* and *Xanks1a*, and the sequence of the *xanks1a* MO oligonucleotide.

Gene		Sequences
*Odc1*	Forward	5′-CCCTATAAGACAAGGAATAC-3′
	Reverse	5′-TCCATTCCGCTCTCCTGAGCAC-3′
*Xanks1a*	Forward	5′-GTCAGATCTCGTCACACTGC-3′
	Reverse	5′-AGACTTGGACAGACTCCTGC-3′
*Xanks1a*-MO	-	5′-AATAACTCCTGTTCTTTCCCCATCC-3′

Fig. 1A shows RT-PCR detection of *anks1a* mRNA from fertilization through tadpole stages (NF 1–40), with *odc1* as the loading control. Transcript is present throughout this interval, indicating that *anks1a* is available during the stages used for morphological scoring. Fig. 1B summarizes injection of translation-blocking *xanks1a*-MO into one-cell-stage embryos. Fig. 1C shows lateral views at stage 35–36: non-injected (NI) embryos retain normal craniofacial and axial morphology, whereas 75 ng, 100 ng and 150 ng MO produce a progressive increase in head reduction, pigmentation defects and axial shortening/curvature, most severe at 150 ng. Fig. 1D aligns the MO sequence with the *xanks1a* 5′ region spanning the ATG start codon (Table 1).

**Fig. 1:**
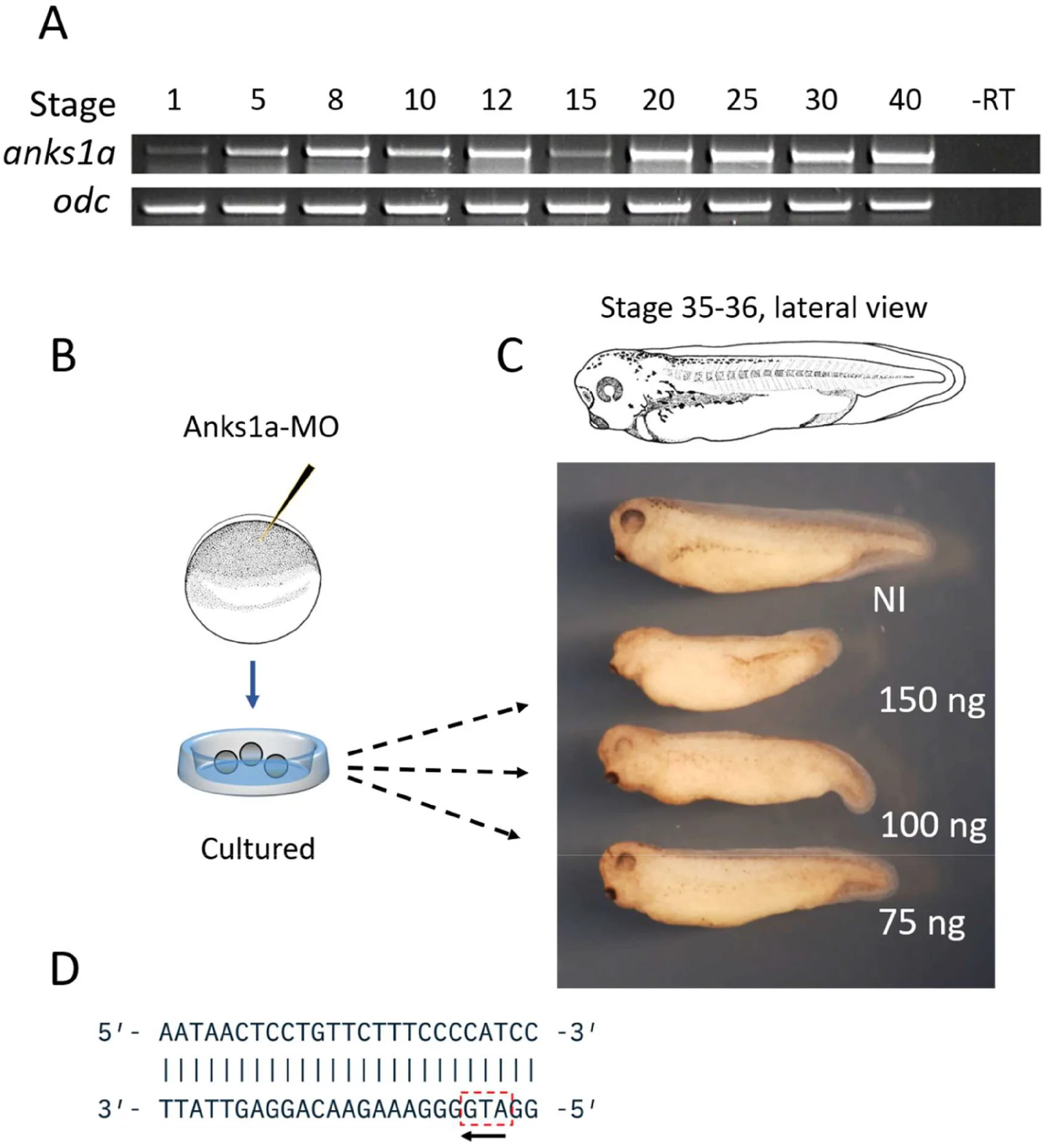
Morpholino knockdown experiment in Xenopus embryos. **(A)** Stage-wise expression of *anks1a* mRNA (RT-PCR). **(B)** Schematic of microinjection of MO oligonucleotides into one cell-stage embryos. **(C)** Lateral views of stage 35–36 embryos showing dose-dependent morphological phenotypes. Non-injected control (NI) and embryos injected with 150 ng, 100 ng, or 75 ng MO are shown. **(D)** Sequence of the *xanks1a* targeting MO oligonucleotide aligned with its complementary DNA sequence from the *anks1a* gene. The ATG start codon is indicated by the red dashed box; 5′→3′ orientation is shown.

Fig. 2 documents the same dose series at stage 42–43 in lateral and ventral/anterior views, using seven numbered landmarks. Relative to NI controls, MO-injected tadpoles show: (1) reduced eye size; (2) reduced or irregular cranial pigment cells; (3) disrupted segmented myotomes; (4) a shortened and/or curved tail; (5) a reduced or malformed cement gland/oral region; (6) reduced or malformed cardiac region; and (7) incomplete or abnormal gut coiling. These defects are visible at 75 ng and become more frequent and more severe at 100 ng and 150 ng. Landmark-level images are qualitative; embryos were not scored as a separate percentage for each of the seven structures.

**Fig. 2:**
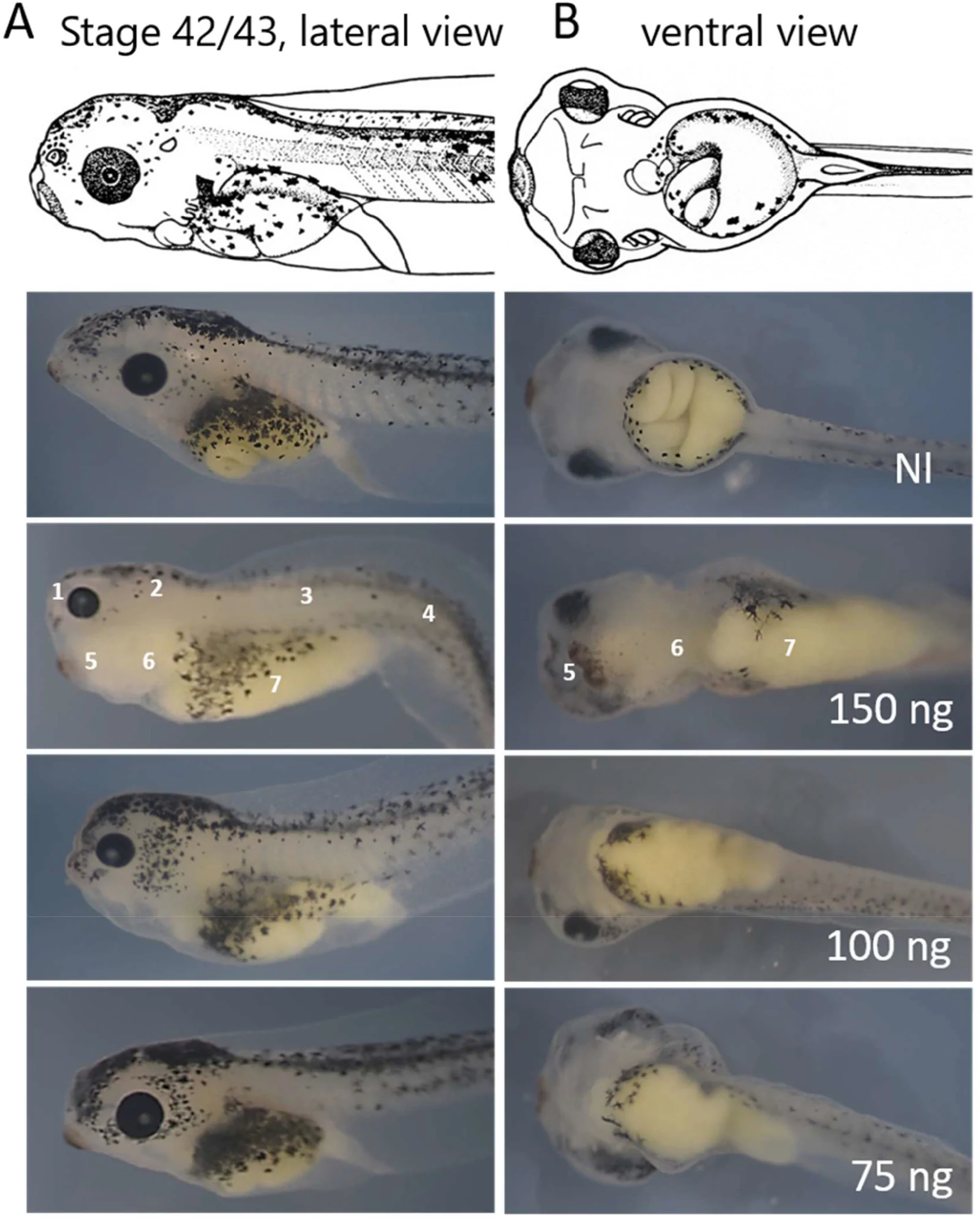
Dose-dependent morphological phenotypes of *anks1a* MO knockdown in *Xenopus* embryos. Top panels show schematic drawings of a stage 42–43 tadpole in (A) lateral (left) and (B) ventral (right) views for orientation. Below, representative live images of non-injected control (NI) and *xanks1a* MO-injected embryos at the indicated doses (150 ng, 100 ng, and 75 ng) are shown in lateral (left column) and ventral/anterior (right column) views. The indicated numbering of the following regions are: 1 eye, 2 pigment cells, 3 segmented myotomes, 4 shortened and curved tail, 5 cement gland or oral region, 6 reduced or malformed cardiac region, 7 gut region.

Fig. 3 tests specificity of the MO phenotypes. At stage 35–36, control embryos were uniformly Normal (100%, *n* = 100). After 150 ng *xanks1a*-MO alone, 3.5% were Normal, 18.8% Mild and 77.6% Severe (3, 16 and 66 embryos; *n* = 85). Co-injection of 150 ng *xanks1a*-MO with 500 pg mouse *ANKS1A-VN* mRNA yielded 16% Normal, 66% Mild and 18% Severe (*n* = 100). The shift from Severe toward Mild/Normal in the co-injection group is the rescue readout; “Rescued” is not used as a scoring class. Mouse *ANKS1A-VN* mRNA lacks the Xenopus morpholino-binding site and is therefore not targeted by *xanks1a*-MO [1]. Raw counts for Fig. 3B are in the Zenodo scoring file [6].

**Fig. 3:**
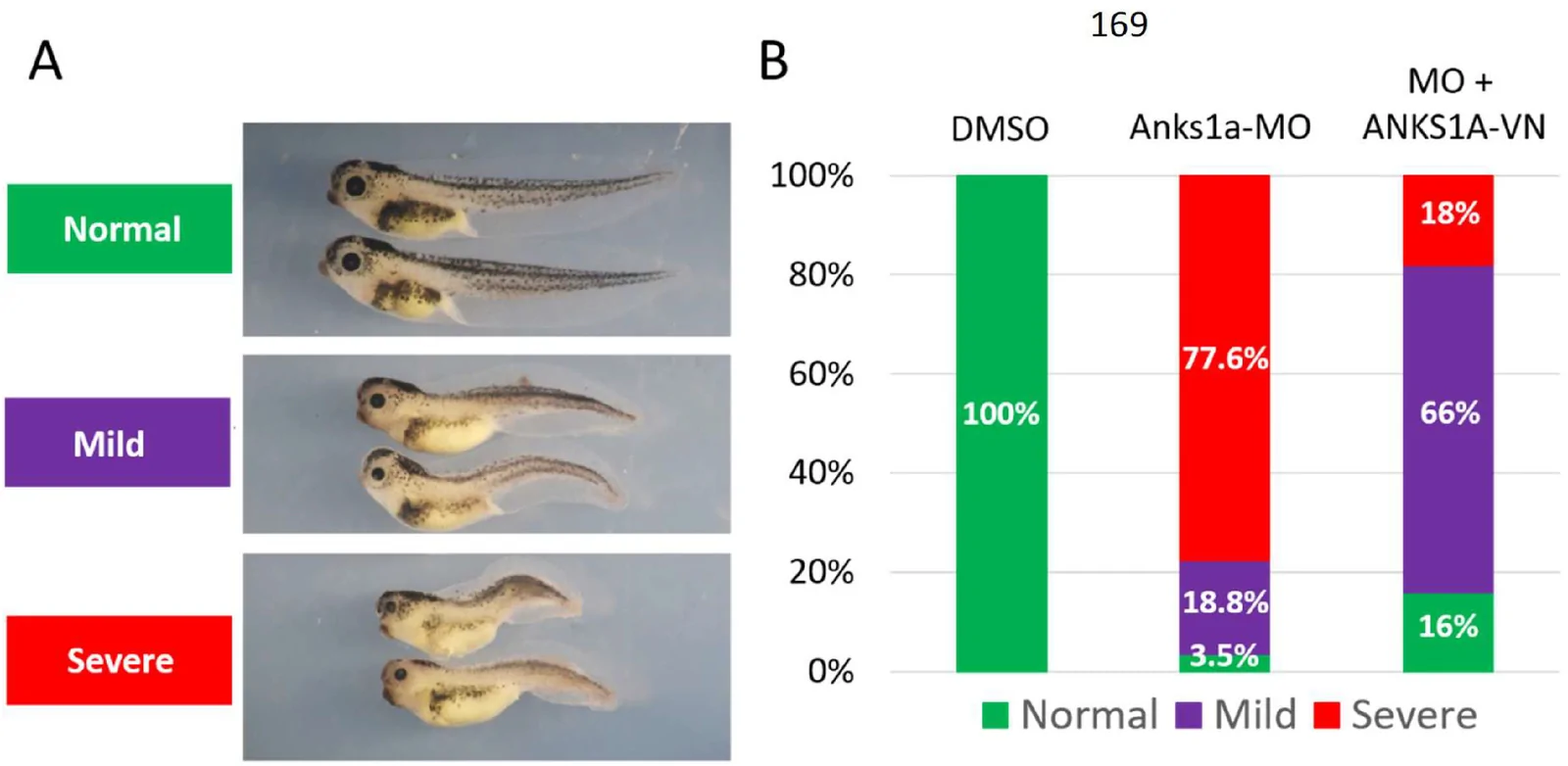
Co-injection of mouse *ANKS1A-VN* mRNA reduces the severity of *xanks1a* MO phenotypes. (A) Representative lateral views of stage 35–36 *Xenopus* embryos. DMSO-injected controls show normal morphology. Embryos injected with 150 ng *xanks1a*-MO display severe developmental defects. Co-injection of 150 ng *xanks1a*-MO with 500 pg mouse *ANKS1A-VN* mRNA partially restores morphology. (B) Percentage of embryos scored Normal, Mild or Severe in the three groups. Percentages are indicated on the stacked bars; class definitions and embryo numbers are given in the Data Description and Methods.

## 4 Experimental Design, Materials and Methods

### 4.1 Embryo collection and culture

Ovulation was induced by injection of 500 units of human chorionic gonadotropin (Sigma-Aldrich) into adult females. Eggs were fertilized in vitro using sperm suspensions prepared from adult males. Fertilized embryos were dejellied in 2% cysteine (pH 7.8–8.0) and thoroughly washed. Embryos were cultured in 30% Marc’s Modified Ringer’s (MMR) solution at 16–18 °C and staged according to the NF normal table. For the present dataset, embryos were grown to stages 35–36 and 42–43 for morphological analysis of whole tadpoles [5].

### 4.2 Morpholino oligonucleotide and mRNA preparation

A translation-blocking antisense MO oligonucleotide targeting *Xenopus anks1a* (*xanks1a*-MO) was synthesized by Gene Tools, LLC. The sequence is 5′-AATAACTCCTGTTCTTTCCCCATCC-3′, which spans the ATG start codon region of *anks1a* mRNA (identical to the MO used previously for loss-of-function studies of Anks1a function in multi-ciliated cells) [1]. Prior to injection, the MO was heated at 55 °C for 5 min and held at 37 °C. Working solutions were prepared at concentrations that delivered 75 ng, 100 ng, or 150 ng per embryo.

For rescue experiments, capped mouse *ANKS1A-VN* mRNA was synthesized in vitro with the mMESSAGE mMACHINE kit (Ambion/Thermo Fisher) according to the manufacturer’s instructions. The mouse transcript does not contain the *Xenopus anks1a* translation-start sequence recognized by *xanks1a*-MO and is therefore MO-resistant. The same heterologous construct was used to rescue *Xenopus anks1a* knockdown in the related primary study [1]. Embryos received 500 pg of this mRNA together with 150 ng *xanks1a*-MO per embryo.

### 4.3 Microinjection

Embryos were injected at the one-cell stage. Working solutions of *xanks1a*-MO were prepared in 10% DMSO in RNA-free distilled water and delivered in 2 nl to give 75, 100 or 150 ng per embryo. Mouse *ANKS1A-VN* mRNA was prepared so that 500 pg was contained in 1 nl. For rescue, 150 ng *xanks1a*-MO and 500 pg mouse *ANKS1A-VN* mRNA were co-injected in 3 nl. Non-injected (NI) embryos were included as untreated controls. Vehicle controls received 2 nl of 10% DMSO in RNA-free distilled water, matching the DMSO content of the MO injectate. After injection, embryos were transferred to 30% MMR and cultured to the desired stages. Dose-response experiments used 75 ng, 100 ng and 150 ng of MO per embryo [1].

### 4.4 RNA isolation and RT-PCR analysis of anks1a expression

To confirm endogenous expression dynamics, total RNA was isolated from staged embryos using RNA-Bee reagent (Tel-Test) and treated with DNase I to eliminate genomic DNA [7]. Reverse transcription was performed with SuperScript II (Invitrogen) using 2 µg of total RNA per reaction [7]. PCR was carried out under the following conditions: 30 s at 94 °C, 30 s at the appropriate annealing temperature, 30 s at 72 °C, for 20–30 cycles. The used primers have listed in the Table 1.

### 4.5 Phenotypic analysis and imaging

Injected and control embryos were allowed to develop to stage 35–36 (early tadpole) and stage 42–43 (swimming tadpole) [4,5]. Live embryos were examined under a stereomicroscope for the following morphological parameters:

• Head size (reduced cranial structures)
• Melanocyte distribution and pigmentation pattern
• Cardiac morphology and cardiac region
• Gut coiling and visceral asymmetry [2]

Phenotypes were classified as Normal, Mild or Severe on the seven Fig. 2 landmarks. Normal: eye size, cranial pigment pattern, cement gland / oral region, reduced or malformed cardiac region, gut coiling, myotomes and tail indistinguishable from stage-matched uninjected embryos. Mild: one or two landmarks abnormal; overall tadpole body plan preserved. Severe: three or more landmarks abnormal, and/or a shortened or curved axis, clearly reduced head or eye, or a clearly reduced or malformed cardiac region or gut coil. Photographic images of live embryos were captured using a Nikon D810 camera (Nikon, Tokyo, Japan) mounted on a stereomicroscope. Images were processed only for brightness/contrast adjustment when necessary; no other digital alterations were applied.

### 4.6 Rescue experiments

To verify specificity of the MO-induced phenotypes, embryos were co-injected with 150 ng *xanks1a*-MO plus 500 pg mouse *ANKS1A-VN* mRNA [1]. Parallel groups of DMSO-injected controls and MO-only embryos were raised under identical conditions. At stages 35–36, embryos were scored and categorized into Normal, Mild, and Severe classes. Percentages of each phenotypic class were calculated and presented as stacked-bar quantifications.

### 4.7 Data recording and quantification

All morphological observations were performed on at least three independent clutches (N ≥ 3). For the stage 35–36 scoring in Fig. 3B, the numbers of embryos were: DMSO, *n* = 100; 150 ng *xanks1a*-MO, *n* = 85; 150 ng *xanks1a*-MO + 500 pg mouse *ANKS1A-VN* mRNA, *n* = 100. Phenotypic frequencies are percentages of these denominators and are deposited with the raw images in Zenodo [6]. Representative images for each dose and rescue condition are shown in Fig. 1, Fig. 2, Fig. 3. These methods extend the *Xenopus* protocols previously described for Anks1a loss-of-function and rescue in multi-ciliated cells, adapted here to whole-embryo morphology at tadpole stages [1,6].

## Limitations

A standard control MO was not included in this dataset. The *xanks1a-*MO and its working dose were those previously optimized for *Xenopus anks1a* knockdown [1]. Possible effects on each Fig. 2 landmark were not scored separately; only whole-embryo Normal, Mild or Severe classes were recorded.

## Ethics Statement

Adult Xenopus laevis were obtained from the Korean Xenopus Resource Center for Research and maintained in approved containers under a 12-h light/dark cycle at 18 °C according to the guidelines of the Institutes of Laboratory Animal Resources. All animal procedures were conducted in accordance with the regulations of the Institutional Animal Care and Use Committee (IACUC) of Hallym University (approval number Hallym-2021-91, 2025-33). Research personnel completed the required educational and training courses for the care and use of laboratory Xenopus.

## CRediT Author Statement

**Vijay Kumar:** Conceptualization, Methodology, Investigation, Data curation, Writing – Original Draft, Visualization. **Shiv Kumar:** Formal analysis. **Unjoo Lee:** Supervision, Formal analysis. **Jaebong Kim:** Conceptualization, Supervision, Funding acquisition, Project administration, Writing – Review & Editing.

## Declaration of Generative AI and AI-assisted Technologies in the Manuscript Preparation Process

During the preparation of this work, the author(s) used Grok for English proofreading. The author(s) reviewed and edited the output as needed and take full responsibility for the content of the published article.
